# Oral Neutrophils: Underestimated Players in Oral Cancer

**DOI:** 10.3389/fimmu.2020.565683

**Published:** 2020-10-09

**Authors:** Maksim Domnich, Jana Riedesel, Ekaterina Pylaeva, Cornelius H. L. Kürten, Jan Buer, Stephan Lang, Jadwiga Jablonska

**Affiliations:** ^1^Department of Otorhinolaryngology, University Hospital Essen, University of Duisburg-Essen, Essen, Germany; ^2^Institute of Medical Microbiology, University of Duisburg-Essen, Essen, Germany

**Keywords:** oral neutrophils, salivary neutrophils, head-and-neck cancer, oral squamous cell carcinoma, salivary diagnostics, oral cancer

## Abstract

The composition of the oral milieu reflects oral health. Saliva provides an environment for multiple microorganisms, and contains soluble factors and immune cells. Neutrophils, which rapidly react on the changes in the microenvironment, are a major immune cell population in saliva and thus may serve as a biomarker for oral pathologies. This review focuses on salivary neutrophils in the oral cavity, their phenotype changes in physiological and pathological conditions, as well as on factors regulating oral neutrophil amount, activation and functionality, with special emphasis on oral cancer and its risk factors.

## Introduction

Head-and-neck cancer (HNC) of the oral cavity (oral squamous cell carcinoma, OSCC), is one of the leading malignancies worldwide (1). OSCC is located in the area of the tongue, upper and lower gingiva, oral floor, palate and buccal mucosa. It can spread to local lymph nodes in the head and neck, while metastasis to distant organs are rare (2). Despite advances in diagnosis and treatment, the 5-year overall survival rate for OSCC remained below 50% for the last three decades (3). Traditional prognostic factors, such as primary tumor size, regional lymph node metastasis, extracapsular spread, surgical margin involvement and perineural invasion are routinely used to predict OSCC outcome (4). In addition to these, a variety of biomarkers are currently under investigation to predict prognosis, allocate treatment and to follow-up responders or recurrences, including but not limited to circulating DNA, exosomes and automated pathology.

Neutrophils are important players in cancer immunology and their in-depth investigation helps to better understand tumor immune escape mechanisms as well as to establish more suitable biomarkers for cancer diagnostics and therapy. Neutrophils are known to contribute to cancer progression or regression via multiple mechanisms, including the suppression of cytotoxic (5) as well as helper (6) T cell responses and the stimulation of tumor angiogenesis (7, 8). Moreover, neutrophils participate in cancer metastasis via formation of premetastatic niche in target organs (9, 10) or via NET-mediated trapping of circulating cancer cells (11). Clinical studies identified blood neutrophil-to-lymphocyte ratio (12) and the number of tumor-infiltrating neutrophils to be negative prognostic factors in a variety of different cancers, including HNC (5, 13).

Here we provide an up-to-date review on oral neutrophils during the development and progression of oral cancer. We discuss tumor-induced systemic changes in circulating polymorphonuclear leukocytes (cPMN), and further alterations that take place in tumor microenvironment. To evaluate possible prognostic role of oral polymorphonuclear leukocytes (oPMN) in HNC, we first address their modifications in healthy oral cavity, and then compare it with their activity in conditions known to be HNC risk factors [aging, smoking, chronic periodontitis (CP)] or with neutrophils associated with oral cancer itself. Finally, we discuss the parallels between the activation status of tumor-associated neutrophils (TANs) and oPMNs, and the potential applicability of these cells in diagnostics.

## Tumor-Induced Alteration of Circulating Neutrophils

Most of the current studies on the prognostic role of neutrophils in cancer focus on the blood neutrophils – PMNs, called also circulating PMNs (cPMNs). And so, it is known that in advanced cancer neutrophilia reflects a systemic inflammatory response to cancer progression (14). Neutrophilia and a high ratio of neutrophils-to-lymphocytes are associated with poor prognosis in many different types of cancer (15), including HNC. These changes could be correlated with both tumor burden and spread to lymph nodes (16). Of note, high PMN levels are associated with worse prognosis in HPV^+^, but not HPV^–^ oropharyngeal cancer patients (17).

The increased numbers of circulating neutrophils may be the result of tumor-induced emergency myelopoiesis (18), but also of prolonged survival of such cells. PMNs of patients with later stages of HNC were shown to have reduced spontaneous apoptosis in comparison to healthy (19). This was probably due to the increased proportion of immature PMNs in circulation of such patients. To the contrary, in another study including patients with oral cancer, circulating PMNs were shown to have elevated apoptosis due to higher caspase-8 activity and elevated activity of TRAIL-mediated mitochondrial cascade, as compared to healthy (20). The surgical removal of the primary tumor partially decreased the predisposition of such PMNs to apoptosis.

Circulating PMNs in HNC show activated status, with reduced expression of CD62L (L-selectin) in neutrophils, as compared to healthy controls (16). The secretion of various cytokines including IL-1b, VEGF, and IL17 was reported to be increased in blood neutrophils in oral cancer, while the secretion of IL-18 and sTRAIL was reduced (21, 22). The ability to release neutrophil extracellular traps (NETs) by PMNs was reported to be significantly enhanced in early stages of HNC, as compared to healthy (23). Moreover, a statistically significant decrease of ROS production by PMNs from HNC patients was observed (19) and it was associated with poor patient prognosis (24).

Importantly, systemic inflammatory response in different types of cancer, including HNC, results in the activation of cPMNs, increasing their cytotoxic response against tumor cells (25). Moreover, progression of cancer leads to the expansion of immature immunosuppressive PMNs (so called granulocytic myeloid-derived suppressor cells) in low density fraction of blood during HNC, lung cancer, or cancers of bladder and ureter (26). Such cells show upregulated CD11b and CD66b expression, suggesting their enhanced degranulation capacity (26, 27). Expansion of such suppressive neutrophils in human HNC is strongly associated with poor survival of patients (28).

## Tumor-Associated Neutrophils

After transmigration into tumor tissue, blood neutrophils undergo dramatic changes of their phenotype and activity, depending on the cytokines and growth factors available in the tumor microenvironment. Multiple reviews are dedicated to the role of tumor-associated neutrophils (TANs) in tumor progression, therefore here we will only briefly address it to provide a link to the key topic of this review – oral neutrophils in cancer. It is known that TANs contribute to tumor vascularization and metastatic spread via the release of VEGF (Vascular endothelial growth factor) and matrix-degrading enzymes, such as MMP9 (7–9). Moreover, such neutrophils are able to modulate adaptive anti-tumor immune responses. On the one hand, TANs express molecules characteristic for antigen-presenting cells (major histocompatibility complex and co-stimulatory molecules) and release stimulatory cytokines that enhance T cell activity (29). On the other hand, the expression of LOX-1 and arginase by neutrophils was reported to be associated with decreased activity and proliferation of effector T cells in tumor tissue (5). The described complexity of neutrophil functions in cancer has not yet been reflected in the clinical studies concerning oral neutrophils.

The prognostic significance of TANs varies between different types of malignancy, indicating the different role of these cells in tumor. For HNC, increased presence of TANs was shown to be a negative, independent prognostic factor for recurrence, as well as overall survival (19).

In HNC, data concerning TANs are limited, possibly due to the minor size of primary tumors. In other cancer types, such as human lung carcinomas, TANs were revealed to have an activated phenotype with high expression of CD11b, CD66b, ICAM-1 and downregulated CD16, in comparison to blood neutrophils. TANs upregulate chemokine receptors responsible for further homing to lymphoid organs (CCR5, CCR7, CXCR3, CXCR4) and downregulate molecules involved in their migration to the tumor site (CD62L, CXCR1, CXCR2). TANs upregulate Fc receptor CD64, death ligand FasL and co-stimulatory molecules CD86, OX40L, 4-1BBl (29). Moreover, they release high amounts of pro-inflammatory cytokines and chemokines that regulate migration of other immune cells into tumor and so influence tumor growth, angiogenesis, and spread. No significant differences in viability, ROS and phagocytosis between cPMN and TANs were observed (29). Due to their expression of co-stimulatory molecules, TANs can efficiently induce proliferation of T cells and their IFNγ production, while blood neutrophils are poor T cell stimulators (29).

PMNs and TANs are already extensively described in HNC context. However, nothing is known about other type of neutrophils that populate environment localized closely to growing oral HNC – oral neutrophils. These cells represent a unique population of neutrophils with phenotypic and functional properties that are distinct from cPMN or neutrophils in other biological compartments, such as mucosa or tumor tissue, due to the specific anatomy and physiology of the oral cavity.

## The Complex Environment of Oral Cavity

Oral cavity represents the first barrier where the external pathogens enter the body and interact with immune defense mechanisms, with saliva as an environment for these interactions. Saliva, secreted by minor and major salivary glands (800–1,500 ml per day, declining during the nighttime) (30–32), is a complex biofluid, containing components from the mucosa, the gingiva crevices, tooth surfaces, nasal secrets and plasma (33–35). Soluble molecules dissolved in saliva are nucleic acids, proteins, mucins/glycoproteins, immunoglobulins, metabolites, drugs and their metabolites (36). The most abundant proteins in saliva are plasma albumin, digestive enzymes and microbicidal proteins, but also variety of hormones, cytokines and chemokines, as well as other molecules with regulatory functions (33). Besides this, saliva contains components with still unknown biological functions (37) that are secreted by exocytosis of the granules of acinar cells in the salivary glands (38). Moreover, saliva contains high amounts of extracellular vesicles, mainly secreted by epithelial cells and salivary glands (39), but also originating from the circulation. Cellular components of saliva include epithelial and immune cells, mostly neutrophils (40).

The mucosal barrier in the oral cavity is considered to be one of the main ecological habitats of the human body (41). Saliva contains bacteria (the most common are *Firmicutes*, *Bacillus*, *Proteobacteria*, *Streptococci*, *Staphylococci*, *Lactobacilli* and *Actinomycetes* species) (42–44), fungi (e.g., *Candida* species) (45), viruses (e.g., Herpes-, Papilloma- and Coxsackieviruses) (46, 47), and other exogenous substances that colonize the mouth and can therefore potentially provide an insight into the relationship of the host with the environment (34). Oral bacterial communities are the second most complex in the body, after the communities of the colon (48). Interestingly, a study of healthy volunteers from 12 locations worldwide have found no significant geographical differences between their salivary microbiota (49). This suggests that the diet and the environment do not significantly influence the composition of the oral microbiome and that the host species is the primary determinant (50). Commensal microbiota play an important role in maintaining oral and systemic health (51), as its presence inhibits colonization by pathogens (“colonization resistance”) (52, 53).

## Oral Neutrophils in Steady State

The contact of the oral cavity with the external environment, the constant presence of normal or invading microbiota as well as secretion of chemokines by activated epithelial cells attract neutrophils. More than 10^11^ neutrophils are produced daily in the bone marrow (54) and are released into circulation to transmigrate into tissues. The trafficking of neutrophils into oral cavity is a constant process, displaying a circadian rhythm, with an increase during the day and a decrease at night (55). Around 10^6^ leukocytes can be isolated after rinsing of the oral cavity. Polymorphonuclear neutrophils represent ca. 83% of cells in such oral rinse, mononuclear cells 17%, and basophils/eosinophils 0.4% (56). Of note, immature forms of neutrophils can also be observed in oral rinse, suggesting direct trafficking from the bone marrow (40). Thus, a redistribution of leukocyte subpopulations in comparison to blood, namely, increased neutrophil and monocyte percentage accompanied by decreased amount of lymphocytes can be observed in oral cavity (57).

The gingival crevices are suggested to be the main point of entry for oPMN into the oral cavity, while only a small proportion of cells originate from salivary glands (55, 58). In agreement, the amount of oPMN in the oral cavity was shown to be significantly decreased in patients without teeth (59, 60). Importantly, there is no correlation between the amount of cPMN and oPMN in healthy individuals (61), which can be explained by a specific local microenvironment, attracting neutrophils to the oral cavity with no impact on the remaining immune system. At the same time, in systemic conditions such as neutropenia or bone marrow transplantation, a shift in numbers of oPMN correlates with cPMN numbers (56, 62).

## The Life-Cycle of Oral Neutrophils

After their release from the bone marrow, neutrophils circulate for 5–9 h and then migrate into tissues where they survive 8–16 h before they die (63). Recently, a prolonged (up to 5 days) survival of activated neutrophils in tissues has been described (64, 65), which ensures the presence of functional neutrophils at the site of inflammation (66). In healthy volunteers the proportion of viable oPMN is significantly lower compared with cPMN, and their maturation more advanced with elevated apoptosis/necrosis (56, 67). Availability of bacteria-derived endotoxin in the oral microenvironment shifts neutrophil fate from apoptosis to necrosis (68, 69). At the same time, oPMN become more resistant to the additional apoptotic stimuli. Unlike cPMN, exudated oPMN are not sensitive to rhTNF-α/cyclohexirnide-induced apoptosis (69). In an elegant study, Hotta et al. demonstrated the lack of sensitivity of oPMN to TNFα-stimulated apoptosis, with lower caspase 3 activity, as compared to cPMN. While in cPMN activation of nuclear factor kB (NF-kB) was induced by TNF-α, in oPMN NF-kB was already activated in steady state, and no further activation was observed by TNF-α treatment. Moreover, no significant effect of NF-kB inhibitor in oPMN was observed (70). This shows that neutrophils become more resistant to apoptotic stimuli during their migration from blood to oral cavity and that this resistance depends on the NF-kB pathway.

## The Phenotypes of Oral Neutrophils

**FIGURE 1:**
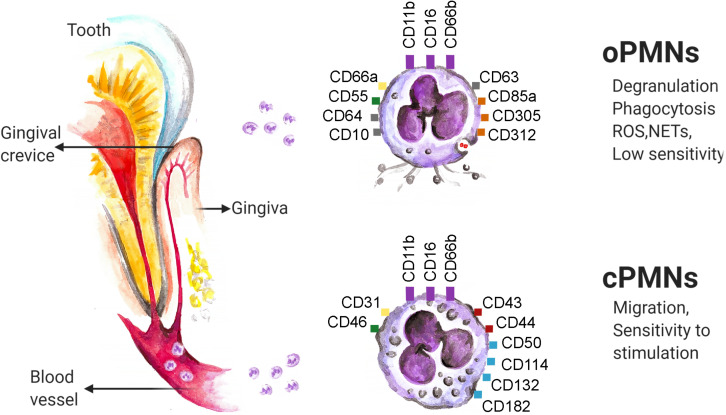
Neutrophils enter the oral cavity through gingival crevice and change their phenotype and properties. Several molecules (namely: CD11b, CD16 and CD66b, marked in violet) are constitutively expressed on neutrophils in all compartments. The variety of molecules responsible for adhesion (namely: CD31, CD66a, marked in yellow), complement-regulation (namely: CD46, CD55 marked in green), regulation of adaptive immunity (namely: CD43 and CD44, marked in red) and intracellular signaling (namely: CD50, CD114, CD132 and CD 182, marked in blue) are down-regulated on oPMNs in comparison to the cPMNs, while the markers of activation (namely: CD10, CD64 and CD 63, marked in gray), complement inhibition (namely: CD55, marked in green) and neutrophil inhibition (namely: CD85a, CD305 and CD312, marked in orange) are up-regulated. ROS, reactive oxygen species; NETs, neutrophil extracellular traps. The data are based on flow cytometry results.

During transmigration from the circulation to the oral cavity, neutrophils are exposed to the new environment, resulting in an activation and dramatic changes of their phenotype and function (Figure 1). Significant modulation of their transcriptome has been shown, with 469 genes downregulated and 119 genes upregulated (71). Majority of these genes are involved in cytokine-cytokine receptor interactions, chemokine signaling pathways, hematopoietic cell lineage development and T cell activation (71).

oPMN express typical neutrophil makers, such as CD11b, CD16, and CD66b. These markers were demonstrated to be constantly expressed on neutrophils, independent of the cell location, level of activation or disease state (72). However, the level of their expression can vary in different conditions (see below). As compared to cPMN, oPMN upregulate molecules reflecting their activation in the oral cavity, including CD63 (a marker associated with degranulation of azurophilic granules), CD66a (adhesion), CD10 (marker of neutrophil differentiation), CD64 (Fc-gamma receptor 1), CD55 (complement regulator) and CD11b (adhesion). Other upregulated molecules in oPMN include inhibitory molecules CD85a, CD305 and CD312 (responsible for interaction with immune cells) (72), while CD16 (Fc-gamma receptor 3) is reduced on oPMN (73). Other molecules responsible for intracellular signaling (CD50, CD114, CD132, CD182) as well as molecules responsible for adhesion (CD31), complement regulation (CD46) or regulation of adaptive immunity (CD43, CD44) are reported to be downregulated on oPMNs (72).

Importantly, two distinct subpopulations of oPMN in healthy donors were described, based on the size and granularity of these cells: para-inflammatory 1 neutrophils with size and granularity comparable to cPMN, and para-inflammatory 2 neutrophils, which are smaller and less granular. These populations exhibit also differences in their function and phenotype, with elevated expression of CD55, CD63 and reduced expression of CD16 and CD170 on para-inflammatory 2 neutrophils, as compared to para-inflammatory 1 (see below) (73).

## The Function of Oral Neutrophils and Its Modulation by the Saliva

Antibacterial functions of neutrophils include adhesion and internalization of bacteria (phagocytosis), production of reactive oxygen species (ROS) that damage membranes and genetical material, the release of neutrophil extracellular traps (NETs) capturing pathogens and the secretion of antibacterial proteins. oPMN isolated from healthy controls demonstrate slightly elevated adhesion to pathogens and significantly higher internalization of bacteria (*A. actinomycetemcomitans*, *P. gingivalis*, *E. coli*) in comparison to cPMN (63). Higher phagocytotic activity in para-inflammatory 2 oral neutrophils was also demonstrated, as compared to para-inflammatory 1 (73). Activation of neutrophils upon phagocytosis (61, 74) led to dramatically increased ROS production by oPMN, but not by cPMN (40). A comparison of both subpopulations of oPMN reveals only slightly increased ROS production by para-inflammatory 2 neutrophils (73). Importantly, the ability to respond to the stimulation with PMA is preserved in both populations in healthy donors (73).

To immobilize and kill distantly localized microorganisms, neutrophils release NETs (75). Importantly, increased NET formation by oPMN, in comparison to cPMN, was reported (63), with para-inflammatory 2 subpopulation showing significantly higher release (73). This was in line with their elevated phagocytosis. In addition to changes in effector function, migratory capacity of neutrophils decreases significantly once they have transmigrated from the bloodstream to the oral cavity. oPMN exhibit random chemotactic movement with a shorter distance as well as decreased fMLP receptor expression (63).

The effect of saliva on neutrophils in the oral cavity is complex and depends on multiple mediators, such as cytokines, chemokines, various proteins or glycoproteins, as well as commensal and pathogenic bacteria and fungi. Several bacterial species are reported to modulate neutrophil functions *in vitro.* The effect of different oral microorganisms, both commensal (*S. oralis*, *S. sanguinis*, *S. salivarius*) and pathogenic (*S. mutans*, *A. actinomycetemcomitans*, *P. gingivalis*), on the activation status of cPMN was described by Oveisi et al. (76). While CD63 and CD11b/CD18 markers were upregulated after exposure to both commensal and pathogenic bacteria, commensal microorganisms in biofilms induced the selective increase of CD66, CD64, CD55, while pathogenic bacteria induced the expression of lipopolysaccharide receptor CD14. Moreover, only commensal bacteria in biofilms stimulated degranulation, phagocytosis, ROS production and NET formation, while pathogenic bacteria showed no effect (76). Coexistence of *F. alocis* with other pathogens induced the secretion of proinflammatory cytokines from epithelial cells and promoted apoptosis of neutrophils (77). This was responsible for increased pathological conditions in oral cavity. Interestingly, *F. alocis* has been shown to be resistant to oxidative stress and to inhibit PMA-induced NET-production. Moreover, this bacterium can survive within neutrophils, repressing their ROS release and maturation of granules. This in turn prolongs neutrophils lifespan and leads to elevated inflammation and tissue damage (77, 78). At the same time, components of bacteria-free saliva (e.g., carbohydrates) limit tissue-damaging neutrophil inflammatory responses (ROS production and release of hydrolytic enzymes) to microbiota (61, 79, 80).

## Risk Factors for Oral Cancer and Their Influence on the Activity of oPMN

**FIGURE 2:**
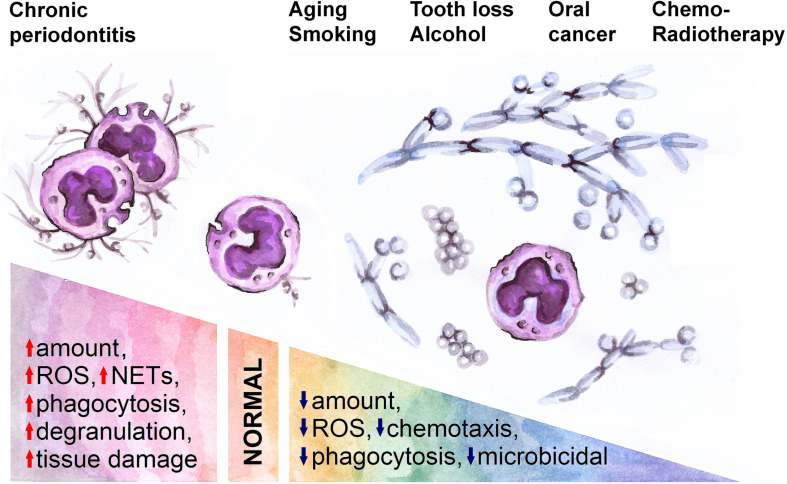
A spectrum of normal and pathological conditions is associated with changes in oral neutrophil activity. Inflammation (chronic periodontitis) in oral cavity activates neutrophils and thus leads to tissue damage, while aging, smoking, tooth loss, alcohol consumption, oral cancer and its treatment are associated with decreased neutrophil activity and expansion of pathological bacteria and fungi in oral cavity. ROS, reactive oxygen species; NETs, neutrophil extracellular traps.

Main risk factors for oral cancer include age (81), tobacco and alcohol consumption (82), and chronic inflammation (e.g., periodontitis) (83, 84). All these factors have also the potential to influence the phenotype, activation and functions of oPMN (Figure 2).

### Aging

Aging is associated with a significant impairment of neutrophil responses in the oral cavity. Elderly individuals (60–85 years old) in comparison to younger persons (20–50 years old) show a reduction of oPMN numbers, which is in line with overall decreasing innate immune responses in elderly (85). Moreover, elevated granulocyte-macrophage colony-stimulating factor (86) in the saliva of aged individuals was reported, which could be responsible for the sensitivity of oPMN to bacteria-induced apoptosis (86). Also, cPMN from the elderly group express lower amounts of CXCR1, CD11b/CD18 integrin and CD62L (L-selectin), which could be responsible for the decreased migration of such cells to the oral cavity (86). Aging is associated with diminished salivary flow rates and reduced production of anti-microbial factors (transferrin and lactoferrin) by oPMN (87). Neutrophil antimicrobial functions, such as phagocytosis, are impaired with age in both, cPMN and oPMN (86). Moreover, aged oPMN show decreased generation of ROS and reduced killing activity (87). All these changes in oPMN functionality could be responsible for the elevated risk for the development of oral neoplasia in elderly.

### Smoking and Alcohol Consumption

The influence of smoking on oral cells is mediated by nicotine as well as a wide range of other accompanying hazardous substances that are included in tobacco smoke. Besides the direct influence, tobacco also affects the pH of saliva (88) and can thus potentially influence the functions of neutrophils. *In vitro* experiments on the effect of nicotine on cPMN demonstrate a disturbed balance between antibacterial and tissue-damaging properties of neutrophils: on the one hand, a dose-dependent suppression of chemotaxis, phagocytosis (89) and diminished ROS-dependent killing is observed (90), on the other hand, such cells show enhanced degranulation and elastase release (89), but their viability is not influenced (89, 90). The data obtained from *in vivo* studies of the effect of smoking on the oPMN functions remain controversial. While some studies show an increased phagocytic activity of oPMN directly after smoking (91), suggesting a direct effect of tobacco smoke on these cells, others demonstrate reduced phagocytic activity and viability of oPMN in smokers, as compared to non-smokers (92, 93). The observed differences may be explained by the different time point of analysis, directly after exposure or chronic changes, or the different impact of various components of the smoke (94), and should still be investigated.

While no data concerning the effect of alcohol consumption on oPMNs and are available, data about cPMNs report the correlation of blood level of gamma-glutamyl transpeptidase (as a measure of alcohol consumption) with compromised neutrophil bacterial killing (95).

### Inflammation in the Oral Cavity

Inflammation is considered to be a hallmark of cancer (96). In agreement, inflammation in oral cavity increases the risk of oral cancer (97). In the inflammatory oral diseases, such as gingivitis or CP, increased amounts of oPMN are observed (55). This can be due to chemoattractants produced by epithelium, but also due to increased oPMN-derived CCL3 or IL-1 (71). Patients with CP show more than a 2.5-fold increase of oral neutrophil counts, as compared to healthy individuals (71). Such neutrophils are of the pro-survival neutrophil phenotype with a prolonged lifespan (71).

The functionality of oPMN reflects the activation of the immune system and may serve a diagnostic parameter for the disease severity and a prognostic marker. In chronic periodontitis (CP) patients distinct changes of the neutrophil transcriptome during migration to the oral cavity has been shown, with 2,386 downregulated and 1,207 upregulated genes in oPMN, compared to cPMN. The major differences were observed in genes responsible for the regulation of apoptosis, but also Toll-like receptor signaling pathways, chemokines and cytokines (71). These changes have an impact on the survival and functions of oPMN.

Inflammatory diseases are often accompanied by the presence of pathogenic bacteria in the oral cavity. Only in rare cases there is one single pathogen inducing the disease, more often it is a shift of microbiome toward certain pathogenic bacteria. In case of CP, the predominance of *Actinomyces* species, which compose much of the supragingival and subgingival plaque microbiota, *P. intermedia*, *Bacteroides species*, and *F. nucleatum* are reported (98, 99). oPMN become activated by invading microorganisms, which contributes to tissue damage and disease progression. Decreased amounts of oPMN together with their suppressed function might be responsible for the development of secondary bacterial or fungal infections in such individuals. The changes in oPMN phenotype and functions are well studied in CP, while in other inflammatory conditions data is still scarce. As compared to the healthy state, CP oPMN gain proinflammatory phenotype, characterized with decreased size and granularity together with prominent activation (upregulation of CD63, CD66a, CD10, CD64, CD55, CD11b/CD18) (73). Lakschevitz et al. reported the upregulation of degranulation (CD63) and adhesion (CD11b, CD66, CD66b, CD66c, CD66e) markers on CP oPMN (72).

Electron microscopy shows elevated phagocytosis (measured as an increase of early and late phagosome counts) and greater degranulation (lower number of granules) of oPMN during CP. This is in line with upregulated expression of CD63 while no differences in granule content is observed (73). The enhanced activation of oPMN is reflected in the induction of myeloperoxidase (MPO) (100). Proinflammatory neutrophils in CP demonstrate elevated ROS production, but in contrast to neutrophils in healthy state, they show no increase of ROS production in response to PMA, suggesting an exhausted phenotype (73). In agreement, NET release estimated by MPO and histone citrullination, is also significantly increased in proinflammatory neutrophils in chronic periodontal disease (73).

Thus, the changes of oPMN functions induced by the contact with pathogens during the course of disease may lead to several unfavorable consequences. On the one hand, suppression of neutrophil functions results in pathogen persistence and spread. On the other hand, hyperactivated neutrophils can cause tissue damage due to the release of proteases (neutrophil elastase or matrix metallopeptidase 9), NETs (101) or pro-inflammatory cytokines, such as IL-1 (71).

As already mentioned, inflammation due to tissue damage is a known cancer hallmark. The presence of activated neutrophils in the oral environment may influence the progression of HNC, as TNFα and IL8 released by neutrophils were shown to increase tumor cell line invasion *in vitro* (102). Thus, the changes of oral neutrophil functions in certain inflammatory conditions (including periodontitis, the known risk factor of oral cancer) may be permissive for cancer development and progression.

## Oral Cancer and Microbiome Shift

Oral cancer is a consequence of the multiple factors present locally in the oral cavity as well as systemically. Inflammation transforms oral ecosystem, including microbiome and immune components, and leads to the formation of premalignant and malignant lesions. Later, growing tumor influences the microenvironment by releasing a wide range of biologically active molecules, such as cytokines, chemokines and growth factors.

An increased predominance of certain bacteria (e.g., *P. gingivalis*, *F. nucleatum*, *P. intermedia*, *C. gingivalis*, *P. melaninogenica*, *S. mitis*, as well as *Veillonella*, *Actinomyces*, *Clostridium*, *Haemophilus* or *Enterobacteriaceae*) correlates strongly with OSCC (99, 103–106). Such association between changes in oral microbiome and the presence of oral cancer can be explained by different causal links: one hypothesis is, that certain bacteria may cause DNA damage in oral epithelium by secreted endotoxins (107) or induce inflammatory responses supporting cancer development (108). On the other hand, changed immune responses in cancer may promote the expansion of pathological microorganisms in the oral cavity. Moreover, tumor-derived molecules solved in saliva serve as chemoattractants and potent regulators of inflammatory cell function (109). Such factors can influence trafficking and activation of immune cells in the oral cavity.

Bacteria are not the only factors in the oral cavity influencing neutrophil properties. The prevalence rate of HPV in normal human mucosa depend from different sociodemographic variables, sexual behavior and sensitivity of the diagnostic techniques (110). HPV is associated with an increased risk of head and neck cancer (HNC), but the prognosis of HPV-positive tumors is better compared to HPV-negative cases (111, 112). It could be demonstrated that HPV-positive OSCC cells contain secondary changes in genes and pathways involved in activation of the host anti-viral interferon signaling (113). As interferons have been shown to have anti-tumoral capacity (114) and to prime anti-tumor phenotype of neutrophils (8, 10, 115), this phenomenon should be further exploited. Overexpression of HPV proteins in OSCC cells is also associated with an impaired neutrophil infiltration to the tumor, possibly due to the downregulated expression of IL-8 (116). While recently a comprehensive single cell RNA sequencing study investigated the differences between intratumoral immune cells isolated from HPV^+^ and HPV^–^ HNC [large differences between B-cells, myeloid cells and conventional CD4^+^ T-cells, rare differences between regulatory CD4^+^ T-cells and CD8^+^ T-cells] (117), there is to the best of our knowledge no study investigating the influence of HPV status on oPMN phenotype and function.

## Changes in Oral Cavity Microenvironment Due to Oral Cancer

Saliva contains various proteins derived from cells populating oral cavity that might attract and activate neutrophils. The levels of such molecules may therefore potentially be used as biomarkers. Saliva contains several chemokines attracting neutrophils. The significant increase of CXCL-8 (118, 119), CXCL-10 and CCL-14 in saliva of patients with head-and-neck carcinoma was reported (120). Moreover, saliva CCL7 levels are shown to correlate positively with lymph node metastasis, tumor size and clinical stage (121). Cytokines and cytokine-coding mRNA in saliva are also shown to be predictors for OSCC progression (51). Goertzen et al. showed that cancer patients have significantly increased pro-inflammatory cytokines, such as IL-1α, IL-1β, IL-6, IL-8 and TNF-α in saliva, as compared to controls (122). Salivary levels of IL-6 (118, 119) may serve as oral cancer predictors (123). Higher levels of growth factors in saliva also correlate with oral inflammation and tumor invasion (37). Importantly, the upregulation of all these factors has a significant role in the activation of neutrophils.

Reduced abundance of peptidyl-prolyl cis-trans isomerase A (PPIA, also known as cyclophilin-A) appeared to be a factor that might predict poor prognosis of OSCC patients (124). This cytosolic molecule being released from the cell, is a potent chemoattractant for neutrophils through the receptor, CD147 (125).

Neutrophil-derived molecules in saliva may reflect the amount and activation status of oPMN. Proteomic analysis of human saliva and saliva-derived extracellular vesicles from healthy individuals and patients with OSCC revealed a significant overrepresentation of proteins related to acute inflammatory response, regulation of humoral response and regulation of hydrogen peroxide metabolic processes (124). Elevated levels of total protein and neutrophil-derived molecules (lysozyme) in saliva were reported for oral cancer patients (126). During severe oral cancer, levels of TNF-α, IL-1 and RANKL are also elevated (33). In neutrophils, MPO makes up to 5% of the total protein content (127). MPO level in saliva increases proportionally to the number of oPMN (100) and therefore is elevated during oral inflammation (128, 129) High levels of neutrophil-derived defensine-1 in saliva can be a sensitive marker for earlier stages of OSCC, while in other conditions, such as glossodynia or oral discomfort, the levels are comparable with healthy controls (130, 131).

Cortisol levels in saliva are significantly increased in OSCC patients in comparison to controls (132), which may potentially influence neutrophil trafficking (133) and functions (134). Thus, saliva from oral cancer patients contains multiple factors regulating oPMN functions or reflecting their activation status during disease progression.

## Oral Neutrophils During the Course of Oral Cancer

Neutrophils present in tumor microenvironment can suppress or potentiate cancer progression (135, 136), depending on their modulation via tumor microenvironment. This can occur locally as well as systemically in distant organs (137). oPMN being in close contact to the tumor site, may influence tumor development, therefore the assessment of their functions may serve an important diagnostic tool. However, only few studies that focus on oPMN in cancer are available.

oPMN isolated from patients with untreated OSCC, demonstrate comparable phagocytic activity, but significantly lower chemotactic capacity to fMLP, as compared to healthy. Moreover, lower superoxide production in response to fMLP and PMA treatment is observed. In agreement, reduced *Candida* killing is observed in such neutrophils (138, 139).

Radio(chemo)therapy is a treatment option for OSCC either in the primary (definitive) setting or as an adjuvant to surgery (2). Irradiation is reported to damage major salivary glands and to impair the salivary flow (140, 141). This in turn is one of the reasons for post-radiation caries and shifts in oral microflora (126, 142). Chemotherapy also has a prominent influence on granulopoiesis in bone marrow, leading to significant neutropenia and bacterial complications (143). The impact of the chemoradiotherapy on oPMN functions was also described, showing suppression of neutrophil chemotaxis, reduced superoxide production and impaired *Candida* killing by oPMN (138). This could be the cause for elevated Candida infections in cancer patients (144, 145).

Neutrophil activity in oral cavity reflect changes in the emergency myelopoiesis in the bone marrow, therefore could be used as prognostic tool in certain conditions (59). In agreement, the increase of oPMN numbers was demonstrated to correlate with successful bone marrow transplantation after immunosuppressive treatment of patients with non-Hodgkin’s lymphoma or multiple myeloma. Importantly, the changes of oPMN counts were observed 1–2 days earlier than in blood (59). Other studies indicated that oPMN counts, rather than cPMN counts, provide better accuracy in prediction of clinical events associated with myelosuppressive chemotherapy-induced neutropenia (e.g., the onset and resolution of fever) (146).

## Oral Neutrophils as Possible Biomarkers

Nevertheless, using neutrophils from blood or tumor tissue as biomarkers has practical and technical limitations, most importantly, there are often only modest changes in neutrophil numbers in the peripheral blood of tumor patients, while tumor biopsies are restricted in size, resulting in challenging analysis or non-representative results.

In contrast, analysis of saliva could offer an alternative route for the evaluation of tumor-induced changes of neutrophil activity, especially in HNC situation. Salivary diagnostics is a non-invasive procedure that offers easier applicability, lower cost and less sensitivity to technical variations than blood draws or tissue analysis (37). Saliva reflects local changes in the oral cavity with higher accuracy than systemic parameters (18), therefore oPMN could have higher prognostic value than cPMN in HNC progression.

Numbers of neutrophils in blood and in tumor are known to correlate with tumor stage and can be predictors for the HNC prognosis (5, 7, 19). As numbers of neutrophils in saliva possibly reflects the emergency granulopoiesis (18) and the presence of tumor-derived chemoattractants in saliva, including CXCL-8 (118, 119), may additionally impact the total amount of oPMN, their numbers in saliva might be considered easily accessible biomarker for tumor progression and prognosis of the disease.

**FIGURE 3:**
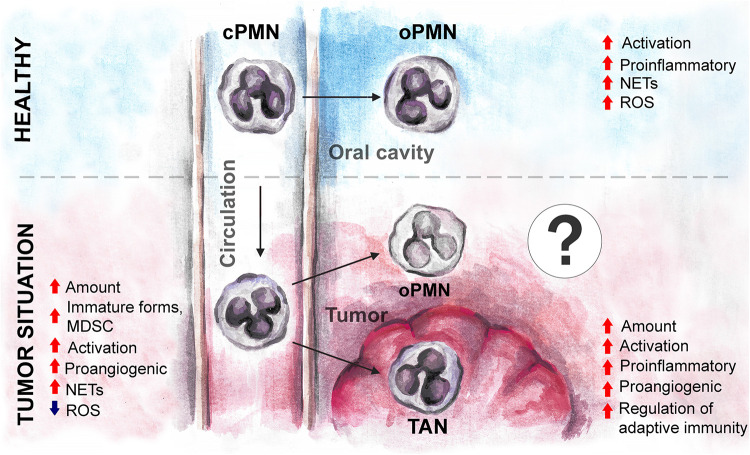
The expected changes in oPMN phenotype and functions under tumor environment in oral cancer. Tumor influences cPMN development and functions before their migration into oral cavity, and may drive the additional changes of oPMN in the local environment, similarly to the changes of tumor-associated neutrophils. cPMN, circulating polymorphonuclear leukocytes; oPMN, oral polymorphonuclear leukocytes; TAN, tumor-associated neutrophils; MDSC, myeloid-derived suppressor cells; ROS, reactive oxygen species; NETs, neutrophil extracellular traps.

While data on oPMN in healthy state or in CP are extensive, the available data about oPMNs in oral cancer are scarce. Taking into consideration high concentrations of cytokines and growth factors released by tumor into oropharyngeal environment and saliva, one could expect the additional activation of oPMN, similar to this described for TANs (Figure 3). The markers considered to be prognostic for HNC and expressed on TANs (such as LOX1) (5), might also be expressed in oPMN and have prognostic significance.

Diminished cytotoxic capacity of oPMNs in certain conditions (aging, smoking) (89, 90) may indicate the lack of cytotoxic activity against tumor cells, thus predisposing to tumor progression. Further studies on oPMN functions in HNC are required to verify this.

Early stages of HNC are characterized with increased NET formation by cPMN. Moreover, tumor-derived factors stimulate production of NETs *in vitro* (23). While oPMNs in healthy are reported to produce high amount of NETs (63), and NET formation is even increased in inflammatory conditions (73), there are no data about NET formation by oPMN in HNC. NETs released by neutrophils contribute to tumor spread (147), thus NET formation by oPMN might also be a useful tool in the evaluation of prognosis in HNC.

## Concluding Remarks

In the recent years, the knowledge about neutrophils and their role in the pathogenesis of various diseases has significantly evolved. Originally characterized as short-living killers, neutrophils are now considered to be important players in the regulation of multiple vital processes. Numerous functions of blood or tumor neutrophils during cancer progression and metastasis have been revealed, including the support of angiogenesis or modulation of the adaptive immune responses. At the same time, very scarce information is available for oral neutrophils.

Here, we are collecting the available evidence that a combination of physiological (aging) and pathological conditions (smoking, oral inflammation) leads to the disbalance of oral neutrophil functions, resulting in the changes in the oral ecosystem. This may contribute to immune evasion and trigger the pro-cancerous mechanisms in the oral cavity. The available data about neutrophils in the oral cavity suggest the suppression of oPMN activity during oral cancer progression, which could lead to microbial complications and chemoradiotherapy side effects. Such changes of oPMN activity might be monitored, providing a useful diagnostic tool for disease progression.

## Author Contributions

MD, JR, EP, and JJ: conceptualization and writing-original draft preparation. MD, JR, EP, CK, JB, SL, and JJ: writing-review and editing. All authors contributed to the article and approved the submitted version.

## Conflict of Interest

The authors declare that the research was conducted in the absence of any commercial or financial relationships that could be construed as a potential conflict of interest.

## References

[1] Bray F Ferlay J Soerjomataram I Siegel RL Torre LA Jemal A. Global cancer statistics 2018: GLOBOCAN estimates of incidence and mortality worldwide for 36 cancers in 185 countries. *CA Cancer J Clin.* (2018) 68:394–424. 10.3322/caac.21492 30207593

[2] Chow LQM. Head and neck cancer. *N Engl J Med.* (2020) 382:60–72. 10.1056/NEJMra1715715 31893516

[3] Kim JW Park Y Roh JL Cho KJ Choi SH Nam SY Prognostic value of glucosylceramide synthase and P-glycoprotein expression in oral cavity cancer. *Int J Clin Oncol.* (2016) 21:883–9. 10.1007/s10147-016-0973-1 27000845

[4] Woolgar JA. Histopathological prognosticators in oral and oropharyngeal squamous cell carcinoma. *Oral Oncol.* (2006) 42:229–39. 10.1016/j.oraloncology.2005.05.008 16150633

[5] Si Y Merz SF Jansen P Wang B Bruderek K Altenhoff P Multidimensional imaging provides evidence for down-regulation of T cell effector function by MDSC in human cancer tissue. *Sci Immunol.* (2019) 4:eaaw9159. 10.1126/sciimmunol.aaw9159 31628161

[6] Castell SD Harman MF Morón G Maletto BA Pistoresi-Palencia MC. Neutrophils which migrate to lymph nodes modulate CD4+ T cell response by a PD-L1 dependent mechanism. *Front Immunol.* (2019) 10:105. 10.3389/fimmu.2019.00105 30761151 PMC6362305

[7] Pylaeva E Harati MD Spyra I Bordbari S Strachan S Thakur BK NAMPT signaling is critical for the proangiogenic activity of tumor-associated neutrophils. *Int J Cancer.* (2019) 144:136–49. 10.1002/ijc.31808 30121947

[8] Jablonska J Leschner S Westphal K Lienenklaus S Weiss S. Neutrophils responsive to endogenous IFN-β regulate tumor angiogenesis and growth in a mouse tumor model. *J Clin Invest.* (2010) 120:1151–64. 10.1172/JCI37223 20237412 PMC2846036

[9] Kaplan RN Riba RD Zacharoulis S Bramley AH Vincent L Costa C VEGFR1-positive haematopoietic bone marrow progenitors initiate the pre-metastatic niche. *Nature.* (2005) 438:820–7. 10.1038/nature04186 16341007 PMC2945882

[10] Wu CF Andzinski L Kasnitz N Kröger A Klawonn F Lienenklaus S The lack of type i interferon induces neutrophil-mediated pre-metastatic niche formation in the mouse lung. *Int J Cancer.* (2015) 137:837–47. 10.1002/ijc.29444 25604426

[11] Cools-Lartigue J Spicer J McDonald B Gowing S Chow S Giannias B Neutrophil extracellular traps sequester circulating tumor cells and promote metastasis. *J Clin Invest.* (2013) 123:3446–58. 10.1172/JCI67484 23863628 PMC3726160

[12] Takenaka Y Oya R Kitamiura T Ashida N Shimizu K Takemura K Prognostic role of neutrophil-to-lymphocyte ratio in head and neck cancer: a meta-analysis. *Head Neck.* (2018) 40:647–55. 10.1002/hed.24986 29076207

[13] Trellakis S Bruderek K Dumitru CA Gholaman H Gu X Bankfalvi A Polymorphonuclear granulocytes in human head and neck cancer: enhanced inflammatory activity, modulation by cancer cells and expansion in advanced disease. *Int J Cancer.* (2011) 129:2183–93. 10.1002/ijc.25892 21190185

[14] Schmidt H Bastholt L Geertsen P Christensen IJ Larsen S Gehl J Elevated neutrophil and monocyte counts in peripheral blood are associated with poor survival in patients with metastatic melanoma: a prognostic model. *Br J Cancer.* (2005) 93:273–8. 10.1038/sj.bjc.6602702 16052222 PMC2361564

[15] Sagiv JY Michaeli J Assi S Mishalian I Kisos H Levy L Phenotypic diversity and plasticity in circulating neutrophil subpopulations in cancer. *Cell Rep.* (2015) 10:562–73. 10.1016/j.celrep.2014.12.039 25620698

[16] Millrud CR Månsson Kvarnhammar A Uddman R Björnsson S Riesbeck K Cardell LO. The activation pattern of blood leukocytes in head and neck squamous cell carcinoma is correlated to survival. *PLoS One.* (2012) 7:e51120. 10.1371/journal.pone.0051120 23251433 PMC3519486

[17] Huang SH Waldron JN Milosevic M Shen X Ringash J Su J Prognostic value of pretreatment circulating neutrophils, monocytes, and lymphocytes in oropharyngeal cancer stratified by human papillomavirus status. *Cancer.* (2015) 121:545–55. 10.1002/cncr.29100 25336438

[18] Sica A Guarneri V Gennari A. Myelopoiesis, metabolism and therapy: a crucial crossroads in cancer progression. *Cell Stress.* (2019) 3:284–94. 10.15698/cst2019.09.197 31535085 PMC6732213

[19] Trellakis S Farjah H Bruderek K Dumitru CA Hoffmann TK Lang S Peripheral blood neutrophil granulocytes from patients with head and neck squamous cell carcinoma functionally differ from their counterparts in healthy donors. *Int J Immunopathol Pharmacol.* (2011) 24:683–93. 10.1177/039463201102400314 21978700

[20] Jabłoñska E Garley M Jabłoñski J. The expressions of intrinsic and extrinsic apoptotic pathway proteins in neutrophils of oral cavity cancer patients: a preliminary study. *Arch Immunol Ther Exp (Warsz).* (2009) 57:229–34. 10.1007/s00005-009-0023-z 19479204

[21] Jablonska E Puzewska W Grabowska Z Jablonski J Talarek LVEGF. IL-18 and NO production by neutrophils and their serum levels in patients with oral cavity cancer. *Cytokine.* (2005) 30:93–9. 10.1016/j.cyto.2004.12.004 15826815

[22] Jablonska E Jablonski J Marcinczyk M Grabowska Z Piotrowski L. The release of soluble forms of TRAIL and DR5 by neutrophils of oral cavity cancer patients. *Folia Histochem Cytobiol.* (2008) 46:177–83. 10.2478/v10042-008-0027-2 18519235

[23] Decker AS Pylaeva E Brenzel A Spyra I Droege F Hussain T Prognostic role of blood NETosis in the progression of head and neck cancer. *Cells.* (2019) 8:946. 10.3390/cells8090946 31438586 PMC6770876

[24] Kaffenberger W Clasen BPE van Beuningen D. The respiratory burst of neutrophils, a prognostic parameter in head and neck cancer? *Clin Immunol Immunopathol.* (1992) 64:57–62. 10.1016/0090-1229(92)90059-W 1606752

[25] Cameron DJ. A comparison of the cytotoxic potential in polymorphonuclear leukocytes obtained from normal donors and cancer patients. *Clin Immunol Immunopathol.* (1983) 28:115–24. 10.1016/0090-1229(83)90194-0 6872356

[26] Brandau S Trellakis S Bruderek K Schmaltz D Steller G Elian M Myeloid-derived suppressor cells in the peripheral blood of cancer patients contain a subset of immature neutrophils with impaired migratory properties. *J Leukoc Biol.* (2011) 89:311–7. 10.1189/jlb.0310162 21106641

[27] Cloke T Munder M Taylor G Müller I Kropf P. Characterization of a novel population of low-density granulocytes associated with disease severity in HIV-1 infection. *PLoS One.* (2012) 7::e48939. 10.1371/journal.pone.0048939 23152825 PMC3496742

[28] Lang S Bruderek K Kaspar C Höing B Kanaan O Dominas N Clinical relevance and suppressive capacity of human myeloid-derived suppressor cell subsets. *Clin Cancer Res.* (2018) 24:4834–44. 10.1158/1078-0432.CCR-17-3726 29914893

[29] Eruslanov EB Bhojnagarwala PS Quatromoni JG Stephen TL Ranganathan A Deshpande C Tumor-associated neutrophils stimulate T cell responses in early-stage human lung cancer. *J Clin Invest.* (2014) 124:5466–80. 10.1172/JCI77053 25384214 PMC4348966

[30] Goel AN Long JL. The oral cavity. *Dysphagia Evaluation and Management in Otolaryngology.* In: Chhetri DK Dewan K editors Amsterdam: Elsevier (2018). p. 5–12. 10.1016/B978-0-323-56930-9.00002-4

[31] Korte DL Kinney J. Personalized medicine: an update of salivary biomarkers for periodontal diseases. *Periodontol 2000.* (2016) 70:26–37. 10.1111/prd.12103 26662480

[32] Bradley RM Fukami H Suwabe T. Neurobiology of the gustatory-salivary reflex. *Chem Senses.* (2005) 30(Suppl):70–1. 10.1093/chemse/bjh118 15738201

[33] Loo JA Yan W Ramachandran P Wong DT. Comparative human salivary and plasma proteomes. *J Dent Res.* (2010) 89:1016–23. 10.1177/0022034510380414 20739693 PMC3144065

[34] Proctor GB. The physiology of salivary secretion. *Periodontol 2000.* (2016) 70:11–25. 10.1111/prd.12116 26662479

[35] Zhao M Yang Y Guo Z Shao C Sun H Zhang Y A comparative proteomics analysis of five body fluids: plasma, urine, cerebrospinal fluid, amniotic fluid, and saliva. *Proteomics Clin Appl.* (2018) 12:1–10. 10.1002/prca.201800008 29781159

[36] Pink R Simek J Vondrakova J Faber E Michl P Pazdera J SALIVA AS A DIAGNOSTIC MEDIUM. *Biomed Pap.* (2009) 153:103–10. 10.5507/bp.2009.017 19771133

[37] Bhat M Bhat D. Salivary diagnostics in oral diseases. *Saliva and Salivary Diagnostics.* In: Gokul S editor. London: Intech open (2019). 10.5772/intechopen.85831

[38] Segawa A Loffredo F Puxeddu R Yamashina S Testa Riva F Riva A. Cell biology of human salivary secretion. *Eur J Morphol.* (2000) 38:237–41. 10.1076/0924-3860(200010)38:4;1-O;FT237 10980674

[39] Berckmans RJ Sturk A Van Tienen LM Schaap MCL Nieuwland R. Cell-derived vesicles exposing coagulant tissue factor in saliva. *Blood.* (2011) 117:3172–80. 10.1182/blood-2010-06-290460 21248061

[40] Yamamoto M Saeki K Utsumi K. Isolation of human salivary polymorphonuclear leukocytes and their stimulation-coupled responses. *Arch Biochem Biophys.* (1991) 289:76–82. 10.1016/0003-9861(91)90444-N 1654849

[41] Keijser BJF Zaura E Huse SM Van Der Vossen JMBM Schuren FHJ Montijn RC Pyrosequencinq analysis of the oral microflora of healthy adults. *J Dent Res.* (2008) 87:1016–20. 10.1177/154405910808701104 18946007

[42] Mark Welch JL Rossetti BJ Rieken CW Dewhirst FE Borisy GG. Biogeography of a human oral microbiome at the micron scale. *Proc Natl Acad Sci USA.* (2016) 113:E791–800. 10.1073/pnas.1522149113 26811460 PMC4760785

[43] Dzidic M Collado MC Abrahamsson T Artacho A Stensson M Jenmalm MC Oral microbiome development during childhood: an ecological succession influenced by postnatal factors and associated with tooth decay. *ISME J.* (2018) 12:2292–306. 10.1038/s41396-018-0204-z 29899505 PMC6092374

[44] Gomez A Espinoza JL Harkins DM Leong P Saffery R Bockmann M Host genetic control of the oral microbiome in health and disease. *Cell Host Microbe.* (2017) 22:269–78.e3. 10.1016/j.chom.2017.08.013 28910633 PMC5733791

[45] Baker JL Bor B Agnello M Shi W He X. Ecology of the oral microbiome: beyond bacteria. *Trends Microbiol.* (2017) 25:362–74. 10.1016/j.tim.2016.12.012 28089325 PMC5687246

[46] Wang J Gao Y Zhao F. Phage-bacteria interaction network in human oral microbiome. *Environ Microbiol.* (2016) 18:2143–58. 10.1111/1462-2920.12923 26036920

[47] Farah CS Balasubramaniam R McCullough MJ. *Contemporary Oral Medicine.* In: Farah CS Balasubramaniam R McCullough MJ editors Cham: Springer International Publishing (2019). 10.1007/978-3-319-72303-7

[48] Kilian M Chapple ILC Hannig M Marsh PD Meuric V Pedersen AML The oral microbiome–an update for oral healthcare professionals. *Br Dent J.* (2016) 221:657–66. 10.1038/sj.bdj.2016.865 27857087

[49] Nasidze I Li J Quinque D Tang K Stoneking M. Global diversity in the human salivary microbiome. *Genome Res.* (2009) 19:636–43. 10.1101/gr.084616.108 19251737 PMC2665782

[50] Wade WG. The oral microbiome in health and disease. *Pharmacol Res.* (2013) 69:137–43. 10.1016/j.phrs.2012.11.006 23201354

[51] Hooper LV Littman DR Macpherson AJ. Interactions between the microbiota and the immune system. *Science.* (2012) 336:1268–73. 10.1126/science.1223490 22674334 PMC4420145

[52] Lorian V. Colonization resistance. *Antimicrob Agents Chemother.* (1994) 38:1693 10.1128/AAC.38.7.1693 PMC284622 7979314

[53] Sullivan Å. Effect of antimicrobial agents on the ecological balance of human microflora. *Lancet Infect Dis.* (2001) 1:101–14. 10.1016/S1473-3099(01)00066-4 11871461

[54] Dancey JT Harker LA Finch CA Dancey JT Deubelbeiss KA Harker LA Neutrophil kinetics in man. find the latest version: neutrophil kinetics in man. *J Clinial Investig.* (1976) 58:705–15. 10.1172/JCI108517 956397 PMC333229

[55] Rindom Schiott C Loe H. The origin and variation in number of leukocytes in the human saliva. *J Periodontal Res.* (1970) 5:36–41. 10.1111/j.1600-0765.1970.tb01835.x 4255144

[56] Rijkschroeff P Loos BG Nicu EA. Oral polymorphonuclear neutrophil contributes to oral health. *Curr Oral Heal Rep.* (2018) 5:211–20. 10.1007/s40496-018-0199-6 30524928 PMC6244624

[57] Sonis ST Mirando DM Lamster IB Stelos P Wilson RE. Evidence suggesting the presence of antigen−antibody complexes on the surface of salivary leukocytes. *J Periodontal Res.* (1979) 14:370–5. 10.1111/j.1600-0765.1979.tb00233.x 161778

[58] Sharry JJ Krasse B. Observations on the origin of salivary leucocytes. *Acta Odontol Scand.* (1960) 18:347–58. 10.3109/00016356009003017

[59] Pink R Vondrakova J Tvrdy P Michl P Pazdera J Faber E Salivary neutrophils level as an indicator of bone marrow engraftment. *Biomed Pap.* (2009) 153:263–9. 10.5507/bp.2009.051 20208965

[60] Rijkschroeff P Loos BG Nicu EA. Impaired polymorphonuclear neutrophils in the oral cavity of edentulous individuals. *Eur J Oral Sci.* (2017) 125:371–8. 10.1111/eos.12367 28833699 PMC5601278

[61] Rijkschroeff P Jansen IDC Van Der Weijden FA Keijser BJF Loos BG Nicu EA. Oral polymorphonuclear neutrophil characteristics in relation to oral health: a cross-sectional, observational clinical study. *Int J Oral Sci.* (2016) 8:191–8. 10.1038/ijos.2016.23 27515277 PMC5113092

[62] Uriarte SM Edmisson JS Jimenez-Flores E. Human neutrophils and oral microbiota: a constant tug-of-war between a harmonious and a discordant coexistence. *Immunol Rev.* (2016) 273:282–98. 10.1111/imr.12451 27558341 PMC5353849

[63] Moonen CGJ Hirschfeld J Cheng L Chapple ILC Loos BG Nicu EA. Oral neutrophils characterized: chemotactic, phagocytic, and neutrophil extracellular trap (NET) formation properties. *Front Immunol.* (2019) 10:635. 10.3389/fimmu.2019.00635 30984197 PMC6449731

[64] Pillay J Den Braber I Vrisekoop N Kwast LM De Boer RJ Borghans JAM In vivo labeling with 2H2O reveals a human neutrophil lifespan of 5.4 days. *Blood.* (2010) 116:625–7. 10.1182/blood-2010-01-259028 20410504

[65] Turner SM Emson CL Hellerstein MK Dale DC. The in vivo half-life of human neutrophils To the editor: anemia in congenital nephrotic syndrome: role of urinary copper and ceruloplasmin loss. *Blood.* (2015) 117:6053–5. 10.1182/blood-2011-02-335844 21636722

[66] Kolaczkowska E Kubes P. Neutrophil recruitment and function in health and inflammation. *Nat Rev Immunol.* (2013) 13:159–75. 10.1038/nri3399 23435331

[67] Fumarulo R Giordano D Laforgia A Larocca A Quarto M. Salivary effects on polymorphonuclear leukocyte functions. *Oral Microbiol Immunol.* (1993) 8:125–7. 10.3109/10.1111/j.1399-302X.1993.tb00558.x 8355986

[68] Crawford JM Wilton JMA Richardson P. Neutrophils die in the gingival crevice, periodontal pocket, and oral cavity by necrosis and not apoptosis. *J Periodontol.* (2000) 71:1121–1129. 10.1902/jop.2000.71.7.1121 10960019

[69] Niwa M Hara A Kanamori Y Kohno K Yoshimi N Mori H Uematsu T Comparison of susceptibility to apoptosis induced by rhTNF-α and cycloheximide between human circulating and exudated neutrophils. *Life Sci.* (1997) 61:205–215. 10.1016/S0024-3205(97)00375-5 9217279

[70] Hotta K Niwa M Hara A Ohno T Wang X Matsuno H The loss of susceptibility to apoptosis in exudated tissue neutrophils is associated with their nuclear factor-κB activation. *Eur J Pharmacol.* (2001) 433:17–27. 10.1016/S0014-2999(01)01480-7 11755130

[71] Lakschevitz FS Aboodi GM Glogauer M. Oral neutrophil transcriptome changes result in a pro-survival phenotype in periodontal diseases. *PLoS One.* (2013) 8:e68983 10.1371/journal.pone.0068983 PMC3708893 23874838

[72] Lakschevitz FS Hassanpour S Rubin A Fine N Sun C Glogauer M. Identification of neutrophil surface marker changes in health and inflammation using high-throughput screening flow cytometry. *Exp Cell Res.* (2016) 342:200–9. 10.1016/j.yexcr.2016.03.007 26970376

[73] Fine N Hassanpour S Borenstein A Sima C Oveisi M Scholey J Distinct oral neutrophil subsets define health and periodontal disease states. *J Dent Res.* (2016) 95:931–8. 10.1177/0022034516645564 27270666

[74] Ashkenazi M Dennison DKA. New method for isolation of salivary neutrophils and determination of their functional activity. *J Dent Res.* (1989) 68:1256–61. 10.1177/00220345890680080901 2576658

[75] Brinkmann V Reichard U Goosmann C Fauler B Uhlemann Y Weiss DS Neutrophil extracellular traps kill bacteria. *Science.* (2004) 303:1532–5. 10.1126/science.1092385 15001782

[76] Oveisi M Shifman H Fine N Sun C Glogauer N Senadheera D Novel assay to characterize neutrophil responses to oral biofilms. *Infect Immun.* (2019) 87:e790–718. 10.1128/IAI.00790-18 30455195 PMC6346121

[77] Miralda I Vashishta A Uriarte SM. Neutrophil interaction with emerging oral pathogens: a novel view of the disease paradigm. In: Belibasakis GN Hajishengallis G Bostanci N Curtis MA editors *Oral Mucosal Immunity and Microbiome.* Cham: Springer International Publishing (2019). p. 165–78. 10.1007/978-3-030-28524-1_12 31732941

[78] Edmisson JS Tian S Armstrong CL Vashishta A Klaes CK Miralda I Filifactor alocis modulates human neutrophil antimicrobial functional responses. *Cell Microbiol.* (2018) 20:1–16. 10.1111/cmi.12829 PMC5980721 29377528

[79] Rijkschroeff P Gunput STG Ligtenberg AJM Veerman ECI Loos BG Nicu EA. Polymorphonuclear neutrophil integrity and functionality are preserved when exposed to saliva. *Arch Oral Biol.* (2018) 92:68–74. 10.1016/j.archoralbio.2018.04.019 29763780

[80] Mangan DF Novak MJ Vora SA Mourad J Kriger PS. Lectinlike interactions of *Fusobacterium nucleatum* with human neutrophils. *Infect Immun.* (1989) 57:3601–11. 10.1128/iai.57.11.3601-3611.1989 2553609 PMC259874

[81] Abrahão R Anantharaman D Gaborieau V Abedi-Ardekani B Lagiou P Lagiou A The influence of smoking, age and stage at diagnosis on the survival after larynx, hypopharynx and oral cavity cancers in Europe: the ARCAGE study. *Int J Cancer.* (2018) 143:32–44. 10.1002/ijc.31294 29405297

[82] Hashibe M Brennan P Benhamou S Castellsague X Chen C Curado MP Alcohol drinking in never users of tobacco, cigarette smoking in never drinkers, and the risk of head and neck cancer: pooled analysis in the international head and neck cancer epidemiology consortium. *J Natl Cancer Inst.* (2007) 99:777–89. 10.1093/jnci/djk179 17505073

[83] Shin YJ Choung HW Lee JH Rhyu IC Kim HD. Association of periodontitis with oral cancer: a case-control study. *J Dent Res.* (2019) 98:526–33. 10.1177/0022034519827565 30779879

[84] Hoare A Soto C Rojas-Celis V Bravo D. Chronic inflammation as a Link between periodontitis and carcinogenesis. *Mediators Inflamm.* (2019) 2019:1029857. 10.1155/2019/1029857 31049022 PMC6458883

[85] Gasparoto TH Vieira NA Porto VC Campanelli AP Lara VS. Differences between salivary and blood neutrophils from elderly and young denture wearers. *J Oral Rehabil.* (2011) 38:41–51. 10.1111/j.1365-2842.2010.02126.x 20663018

[86] Gasparoto TH Vieira NA Porto VC Campanelli AP Lara VS. Ageing exacerbates damage of systemic and salivary neutrophils from patients presenting Candida-related denture stomatitis. *Immun Ageing.* (2009) 6:3. 10.1186/1742-4933-6-3 19327169 PMC2669447

[87] Tanida T Ueta E Tobiume A Hamada T Rao F Osaki T. Influence of aging on candidal growth and adhesion regulatory agents in saliva. *J Oral Pathol Med.* (2001) 30:328–35. 10.1034/j.1600-0714.2001.300602.x 11459318

[88] Rehan F Khan RS Khurshid Z Memon MS Naqvi S Zafar MS. Analysis of resting mouth salivary flow rate and salivary pH of tobacco chewers and smokers. *J Pak Dent Assoc.* (2016) 25:158–63.

[89] Seow WK Thong YH Nelson RD Macfarlane GD Herzberg MC. Nicotine-induced release of elastase and eicosanoids by human neutrophils. *Inflammation.* (1994) 18:119–27. 10.1007/BF01534553 8070897

[90] Pabst MJ Pabst KM Collier JA Coleman TC Lemons-Prince ML Godat MS Inhibition of neutrophil and monocyte defensive functions by nicotine. *J Periodontol.* (1995) 66:1047–55. 10.1902/jop.1995.66.12.1047 8683417

[91] Numabe Y Ogawa T Kamoi H Kiyonobu K Sato S Kamoi K Phagocytic function of salivary PMN after smoking or secondary smoking. *Ann Periodontol.* (1998) 3:102–7. 10.1902/annals.1998.3.1.102 9722694

[92] Archana MS Bagewadi A Keluskar V. Assessment and comparison of phagocytic function and viability of polymorphonuclear leukocytes in saliva of smokers and non-smokers. *Arch Oral Biol.* (2015) 60:229–33. 10.1016/j.archoralbio.2014.09.018 25463900

[93] Sato J Takahashi I Umeda T Matsuzaka M Danjyo K Tsuya R Effect of alcohol drinking and cigarette smoking on neutrophil functions in adults. *Luminescence.* (2011) 26:557–64. 10.1002/bio.1270 21433278

[94] White PC Hirschfeld J Milward MR Cooper PR Wright HJ Matthews JB Cigarette smoke modifies neutrophil chemotaxis, neutrophil extracellular trap formation and inflammatory response-related gene expression. *J Periodontal Res.* (2018) 53:525–35. 10.1111/jre.12542 29574730

[95] Khocht A Schleifer S Janal M Keller S. Neutrophil function and periodontitis in alcohol-dependent males without medical disorders. *J Int Acad Periodontol.* (2013) 15:68–74. 24079098

[96] Murata M. Inflammation and cancer. *Environ Health Prev Med.* (2018) 23:50. 10.1186/s12199-018-0740-1 30340457 PMC6195709

[97] Hooper SJ Wilson MJ Crean SJ. Exploring the link between microorganisms and oral cancer: a systematic review of the literature. *Head Neck.* (2009) 31:1228–39. 10.1002/hed.21140 19475550

[98] Sampaio-Maia B Caldas IM Pereira ML Pérez-Mongiovi D Araujo R. *The Oral Microbiome in Health and Its Implication in Oral and Systemic Diseases.* Amsterdam: Elsevier Ltd (2016). 10.1016/bs.aambs.2016.08.002 27926431

[99] Atanasova KR Yilmaz O. Looking in the *Porphyromonas gingivalis* cabinet of curiosities: the microbium, the host and cancer association. *Mol Oral Microbiol.* (2014) 29:55–66. 10.1111/omi.12047 24506890 PMC3949145

[100] Thomas EL Jefferson MM Joyner RE Cook GS King CC. Leukocyte myeloperoxidase and salivary lactoperoxidase: identification and quantitation in human mixed saliva. *J Dent Res.* (1994) 73:544–55. 10.1177/00220345940730021001 8120219

[101] Pylaeva E Bordbari S Spyra I Decker AS Häussler S Vybornov V Detrimental effect of type I IFNs during acute lung infection with *Pseudomonas aeruginosa* is mediated through the stimulation of neutrophil NETosis. *Front Immunol.* (2019) 10:2190. 10.3389/fimmu.2019.02190 31572395 PMC6749149

[102] Glogauer JE Sun CX Bradley G Magalhaes MAO. Neutrophils increase oral squamous cell carcinoma invasion through an invadopodia-dependent pathway. *Cancer Immunol Res.* (2015) 3:1218–26. 10.1158/2326-6066.CIR-15-0017 26112922

[103] Katz J Onate MD Pauley KM Bhattacharyya I Cha S. Presence of Porphyromonas gingivalis in gingival squamous cell carcinoma. *Int J Oral Sci.* (2011) 3:209–15. 10.4248/IJOS11075 22010579 PMC3469978

[104] Nagy KN Sonkodi I Szöke I Nagy E Newman HN. The microflora associated with human oral carcinomas. *Oral Oncol.* (1998) 34:304–8. 10.1016/S1368-8375(98)00008-6 9813727

[105] Mager DL Haffajee AD Delvin PM Norris CM Posner MR Goodson JM. The salivary microbiota as a diagnostic indicator of oral cancer: a descriptive, non-randomized study of cancer-free and oral squamous cell carcinoma subjects. *J Transl Med.* (2005) 3:1–8. 10.1186/1479-5876-3-27 15987522 PMC1226180

[106] Hu X Zhang Q Hua H Chen F. Changes in the salivary microbiota of oral leukoplakia and oral cancer. *Oral Oncol.* (2016) 56:e6–8. 10.1016/j.oraloncology.2016.03.007 27026576

[107] Mani V Weber TE Baumgard LH Gabler NK. Growth and development symposium: endotoxin, inflammation, and intestinal function in livestock. *J Anim Sci.* (2012) 90:1452–65. 10.2527/jas.2011-4627 22247110

[108] Hanahan D Weinberg RA. Hallmarks of cancer: the next generation. *Cell.* (2011) 144:646–74. 10.1016/j.cell.2011.02.013 21376230

[109] Lin W Karin M. A cytokine-mediated link between innate immunity, inflammation, and cancer. *J Clin Invest.* (2007) 117:1175–83. 10.1172/JCI31537 17476347 PMC1857251

[110] Candotto V Lauritano D Nardone M Baggi L Arcuri C Gatto R HPV infection in the oral cavity: epidemiology, clinical manifestations and relationship with oral cancer. *ORAL Implantol.* (2017) 10:209–20. 10.11138/orl/2017.10.3.209 29285322 PMC5735384

[111] Chaturvedi AK Engels EA Pfeiffer RM Hernandez BY Xiao W Kim E Human papillomavirus and rising oropharyngeal cancer incidence in the United States. *J Clin Oncol.* (2011) 29:4294–301. 10.1200/JCO.2011.36.4596 21969503 PMC3221528

[112] Gillison ML. Evidence for a causal association between Human Papillomavirus and a subset of head and neck cancers. *J Natl Cancer Inst.* (2000) 92:709–20. 10.1093/jnci/92.9.709 10793107

[113] Gillison ML Akagi K Xiao W Jiang B Pickard RKL Li J Human papillomavirus and the landscape of secondary genetic alterations in oral cancers. *Genome Res.* (2019) 29:1–17. 10.1101/gr.241141.118 30563911 PMC6314162

[114] Martin-Hijano L Sainz B. The interactions between cancer stem cells and the innate interferon signaling pathway. *Front Immunol.* (2020) 11:526. 10.3389/fimmu.2020.00526 32296435 PMC7136464

[115] Andzinski L Kasnitz N Stahnke S Wu CF Gereke M Von Köckritz-Blickwede M Type i IFNs induce anti-tumor polarization of tumor associated neutrophils in mice and human. *Int J Cancer.* (2016) 138:1982–93. 10.1002/ijc.29945 26619320

[116] Li C Zhao L Wang Q Ma S Sun J Ma C Neutrophils infiltration and its correlation with human papillomavirus status in the oral squamous cell carcinoma. *Cancer Manag Res.* (2019) 11:5171–85. 10.2147/CMAR.S202465 31239772 PMC6557188

[117] Cillo AR Kürten CHL Tabib T Qi Z Onkar S Wang T Immune landscape of viral- and carcinogen-driven head and neck cancer. *Immunity.* (2020) 52:183–99.e9. 10.1016/j.immuni.2019.11.014 31924475 PMC7201194

[118] St. John MAR Li Y Zhou X Denny P Ho CM Montemagno C Interleukin 6 and interleukin 8 as potential biomarkers for oral cavity and oropharyngeal squamous cell carcinoma. *Arch Otolaryngol Head Neck Surg.* (2004) 130:929–35. 10.1001/archotol.130.8.929 15313862

[119] Sahibzada HA Khurshid Z Khan RS Naseem M Siddique KM Mali M Salivary IL-8, IL-6 and TNF-α as potential diagnostic biomarkers for oral cancer. *Diagnostics.* (2017) 7:21. 10.3390/diagnostics7020021 28397778 PMC5489941

[120] Michiels K Schutyser E Conings R Lenaerts JP Put W Nuyts S Carcinoma cell-derived chemokines and their presence in oral fluid. *Eur J Oral Sci.* (2009) 117:362–8. 10.1111/j.1600-0722.2009.00644.x 19627345

[121] Zhang W Luo J Dong X Zhao S Hao Y Peng C Salivary microbial dysbiosis is associated with systemic inflammatory markers and predicted oral metabolites in non-small cell lung cancer patients. *J Cancer.* (2019) 10:1651–62. 10.7150/jca.28077 31205521 PMC6548009

[122] Goertzen C Mahdi H Laliberte C Meirson T Eymael D Gil-Henn H Oral inflammation promotes oral squamous cell carcinoma invasion. *Oncotarget.* (2018) 9:29047–63. 10.18632/oncotarget.25540 30018735 PMC6044370

[123] Saxena S Sankhla B Sundaragiri K Bhargava AA. Review of salivary biomarker: a tool for early oral cancer diagnosis. *Adv Biomed Res.* (2017) 6:90. 10.4103/2277-9175.211801 28828341 PMC5549541

[124] Winck FV Ribeiro ACP Domingues RR Ling LY Riaño-Pachón DM Rivera C Insights into immune responses in oral cancer through proteomic analysis of saliva and salivary extracellular vesicles. *Sci Rep.* (2015) 5:1–13. 10.1038/srep16305 PMC4633731 26538482

[125] Arora K Gwinn WM Bower MA Watson A Okwumabua I MacDonald HR Extracellular cyclophilins contribute to the regulation of inflammatory responses. *J Immunol.* (2005) 175:517–22. 10.4049/jimmunol.175.1.517 15972687 PMC2862457

[126] Brown LR Dreizen S Handler S Johnston DA. Effect of radiation-induced xerostomia on human oral microflora. *J Dent Res.* (1975) 54:740–50. 10.1177/00220345750540040801 1099136

[127] Bos A Wever R Roos D. Characterization and quantification of the peroxidase in human monocytes. *BBA Enzymol.* (1978) 525:37–44. 10.1016/0005-2744(78)90197-3 28769

[128] Smith AJ Smith G Bash MK Walsh TF. Changes in salivary peroxidase activity observed during experimentally−induced gingivitis. *J Clin Periodontol.* (1984) 11:373–8. 10.1111/j.1600-051X.1984.tb01335.x 6086728

[129] Venezie RD Jenzano JW Lundblad RL. Differentiation of myeloperoxidase and glandular peroxidase in biological fluids: application to human saliva. *J Clin Lab Anal.* (1991) 5:57–9. 10.1002/jcla.1860050111 1847970

[130] Kaur J Jacobs R Huang Y Salvo N Politis C. Salivary biomarkers for oral cancer and pre-cancer screening: a review. *Clin Oral Investig.* (2018) 22:633–40. 10.1007/s00784-018-2337-x 29344805

[131] Mizukawa N Sugiyama K Ueno T Mishima K Takagi S Sugahara T. Defensin-1, an antimicrobial peptide present in the saliva of patients with oral diseases. *Oral Dis.* (2008) 5:139–42. 10.1111/j.1601-0825.1999.tb00078.x 10522210

[132] Bernabé DG Tamae AC Miyahara GI Sundefeld MLM Oliveira SP Biasoli ÉR. Increased plasma and salivary cortisol levels in patients with oral cancer and their association with clinical stage. *J Clin Pathol.* (2012) 65:934–9. 10.1136/jclinpath-2012-200695 22734006

[133] Ince LM Weber J Scheiermann C. Control of leukocyte trafficking by stress-associated hormones. *Front Immunol.* (2019) 10:3143. 10.3389/fimmu.2018.03143 30687335 PMC6336915

[134] Keresztes M Horváth T Ocsovszki I Földesi I Serfozo G Boda K ACTH- and cortisol-associated neutrophil modulation in coronary artery disease patients undergoing stent implantation. *PLoS One.* (2013) 8:e71902. 10.1371/journal.pone.0071902 23967262 PMC3743772

[135] Fridlender ZG Sun J Kim S Kapoor V Cheng G Ling L Polarization of tumor-associated neutrophil phenotype by TGF-β: “N1” versus “N2”. TAN. *Cancer Cell.* (2009) 16:183–94. 10.1016/j.ccr.2009.06.017 19732719 PMC2754404

[136] Granot Z Jablonska J. Distinct functions of neutrophil in cancer and its regulation. *Mediators Inflamm.* (2015) 2015:701067. 10.1155/2015/701067 26648665 PMC4663337

[137] Coffelt SB Wellenstein MD De Visser KE. Neutrophils in cancer: neutral no more. *Nat Rev Cancer.* (2016) 16:431–46. 10.1038/nrc.2016.52 27282249

[138] Ueta E Osaki T Yoneda K Yamamoto T Umazume M. Influence of inductive chemoradiotherapy on salivary polymorphonuclear leukocyte (SPMN) functions in oral cancer. *J Oral Pathol Med.* (1994) 23:418–22. 10.1111/j.1600-0714.1994.tb00088.x 7823303

[139] Ueta E Osaki T Yoneda K Yamamoto T. Functions of salivary polymorphonuclear leukocytes (SPMNs) and peripheral blood polymorphonuclear leukocytes (PPMNs) from healthy individuals and oral cancer patients. *Clin Immunol Immunopathol.* (1993) 66:272–8. 10.1006/clin.1993.1036 8381737

[140] Ben-Aryeh H Gutman D Szargel R Laufer D. Effects of irradiation on saliva in cancer patients. *Int J Oral Surg.* (1975) 4:205–10. 10.1016/S0300-9785(75)80027-5 811576

[141] Marks JE Davis CC Gottsman VL Purdy JE Lee F. The effects of radiation on parotid salivary function. *Int J Radiat Oncol.* (1981) 7:1013–9. 10.1016/0360-3016(81)90152-8 7298398

[142] Cowman RA Baron SS Glassman AH Davis ME Strosberg AM. Changes in protein composition of saliva from radiation-induced xerostomia patients and its effect on growth of Oral *Streptococci*. *J Dent Res.* (1983) 62:336–40. 10.1177/00220345830620030601

[143] Schirm S Engel C Loeffler M Scholz M. Modelling chemotherapy effects on granulopoiesis. *BMC Syst Biol.* (2014) 8:138. 10.1186/s12918-014-0138-7 25539928 PMC4302124

[144] Diaz PI Hong BY Dupuy AK Choquette L Thompson A Salner AL Integrated analysis of clinical and microbiome risk factors associated with the development of oral candidiasis during cancer chemotherapy. *J Fungi.* (2019) 5:49. 10.3390/jof5020049 31200520 PMC6617088

[145] Bertolini M Ranjan A Thompson A Diaz PI Sobue T Maas K Candida albicans induces mucosal bacterial dysbiosis that promotes invasive infection. *PLoS Pathog.* (2019) 15:e1007717. 10.1371/journal.ppat.1007717 31009520 PMC6497318

[146] Akpek G Knight RD Wright DG. Use of oral mucosal neutrophil counts to detect the onset and resolution of profound neutropenia following high-dose myelosuppressive chemotherapy. *Am J Hematol.* (2003) 72:13–9. 10.1002/ajh.10250 12508262

[147] Zhang LM Chen JH. Progression of NETs correlating with tumor-related diseases. *Asian Pacific J Cancer Prev.* (2015) 16:7431–4. 10.7314/APJCP.2015.16.17.7431 26625739

